# Physiological Responses of Wheat Seedlings to Soil Waterlogging Applied after Treatment with Selective Herbicide

**DOI:** 10.3390/plants10061195

**Published:** 2021-06-11

**Authors:** Zornitsa Katerova, Iskren Sergiev, Dessislava Todorova, Elena Shopova, Ljudmila Dimitrova, Liliana Brankova

**Affiliations:** Department Regulators of Plant Growth and Development, Institute of Plant Physiology and Genetics, Bulgarian Academy of Sciences, Acad G. Bonchev Str., Bl. 21, 1113 Sofia, Bulgaria; zkaterova.landzhova@gmail.com (Z.K.); dessita@bio21.bas.bg (D.T.); kostei@abv.bg (E.S.); dim.lyudmila@gmail.com (L.D.); lbrankova@abv.bg (L.B.)

**Keywords:** antioxidants, herbicide, stress markers, waterlogging, wheat

## Abstract

Waterlogging impairs crop development and considerably affects plant productivity worldwide. Wheat is sensitive to waterlogging. Serrate^®^ (Syngenta) is a selective herbicide controlling annual grass and broadleaf weeds for use in wheat. To extend the existing information about the physiological effects of selective herbicides (Serrate^®^ in particular) and subsequent waterlogging in wheat, we monitored phenotype alterations and examined key enzymatic and non-enzymatic antioxidant defense systems together with typical oxidative stress biomarkers. Seventeen-day-old wheat (*Triticum asetivum* L., cv. Sadovo-1) plants were sprayed with Serrate^®^; 72 h later, waterlogging was applied for 7 days, and then seedlings were left to recover for 96 h. The herbicide did not alter plant phenotype and increased antioxidant defense, along with H_2_O_2_ content, confirming the wheat’s tolerance to Serrate^®^. Evident yellowing and wilting of the leaves were observed at 96 h of recovery in waterlogged wheat, which were stronger in plants subjected to Serrate^®^ + waterlogging. Waterlogging alone and herbicide + waterlogging gradually enhanced the content of stress markers (malondialdehyde, proline, and H_2_O_2_), non-enzymatic antioxidants (low-molecular thiols and total phenolics), and the activity of superoxide dismutase, guaiacol peroxidase, and glutathione reductase. The effects of herbicide + waterlogging were stronger than those of waterlogging alone even during recovery, suggesting that Serrate^®^ interacted synergistically with the subsequently applied flooding.

## 1. Introduction

Different climate models predict that negative environmental alterations will expand due to climate change driven by pollution and global warming, and the reductions in plant productivity will become much more noticeable [1,2,3,4]. Waterlogging is a substantial obstacle to sustainable agriculture and is expected to increase due to climate change. It can be a result of soil erosion; bad soil drainage; or unexpected, sudden, and heavy rainfall leading to floods [5]. Excess water negatively affects plant growth and development, leading to sizable yield losses. Waterlogging causes a number of changes in important soil physiochemical properties such as pH, redox potential, and oxygen access. Thus, plants grown on waterlogged soil are subject to adverse growth and negative developmental conditions such as hypoxia (O_2_ insufficiency) or anoxia (lack of O_2_), inhibition of aerobic respiration, energy deprivation, and oxidative stress. These conditions trigger an over-accumulation of reactive oxygen species (ROS), which impede plant growth, leading to senescence and cell death. In wheat production, significant harvest losses due to waterlogging were estimated to be 15–20% on an annual basis [6,7,8,9]. 

Another major problem in crop cultivation is spontaneous weed growth. The necessity for control of wild plants in arable fields led to the development of different chemicals to selectively demolish weeds, increase crop yields, and economize human resources and time. Currently, herbicide usage plays an important role in commercial agriculture. Through their mechanism of action, herbicides can be classified into four major categories: affecting photosynthesis or photosynthesis-related pigments, auxin-type, inhibitors of amino acid biosynthesis, and inhibitors of fatty acid biosynthesis. Herbicides can be grouped as selective (which affect certain types of plant species) or total (which affect all plant species) due to plant sensitivity.

The unique composition of Serrate^®^ (Syngenta), containing clodinafop-propargyl (prop-2-ynyl(R)-2-[4-(5-chloro-3-fluoro-2-pyridyloxy)phenoxy]propionate, an inhibitor of fatty acid biosynthesis) and pyroxsulam (([*N*-(5,7-dimethoxy[1,2,4]triazolo [1,5-a]pyrimidin-2-yl)-2-methoxy-4-(trifluoromethyl)pyridine-3-sulfonamide], an inhibitor of amino acid biosynthesis), as well as a herbicide safener (cloquintocet-mexyl, (RS)-1-methylhexyl [(5-chloro-8-quinolyl)oxy]acetate), causes its particular effectiveness in cereal crop protection by controlling annual grass and broadleaf weeds [10]. Serrate^®^ is systemic and selective for wheat, rye, and triticale, which are tolerant to its composition.

In general, plants under stress activate endogenous antioxidant defense systems to counteract the harmful effects of the stressor with the activation of diverse transcription factors and phytohormones, and the participation of diverse enzymatic and non-enzymatic antioxidants [11,12]. Due to their rooted way of life, plants are naturally exposed to more than one stress factor, occurring sequentially or simultaneously. There is a small number of studies on waterlogging stress combinations, mainly considering biotic stress or priming strategies as a second factor ([13,14,15,16,17]. In some cases, plants may adapt due to their plasticity and evolved complex system of sensing, signaling, and responding adequately to stressors [11,12]. The response systems include phytohormones, the gene-metabolic network, antioxidant defense (enzymatic and non-enzymatic), etc. In other cases, plants are unable to adapt as the second stressor enhances the negative impact of the first stress factor and leads to programmed cell death [2,11]. Despite the known negative effects on plants, ROS may function by signaling molecules that affect transcription and translation. Their role is complex and unique for each cell compartment, for each single stressor, and for each stress combination (discussed in [11,18,19]). The balance between antioxidant activation and ROS formation is essential for plants subjected to oxidative stress events and determines the output effect on a plant’s ability to survive (or not survive) the oxidative stress. 

To the best of our knowledge, research on the effect of selective herbicide (Serrate^®^ in particular) and subsequent waterlogging has not been reported, especially for wheat culture. To fill this gap, we examined the effects of selective herbicide Serrate^®^ and consequent waterlogging of soil on wheat physiological responses. The current study aimed to assess the additive effect of herbicide and waterlogging by monitoring phenotype alterations and the examination of basic enzymatic and non-enzymatic antioxidant defense systems together with typical oxidative stress biomarkers.

## 2. Results

### 2.1. Effect on Wheat Phenotype 

**Figure 1:**
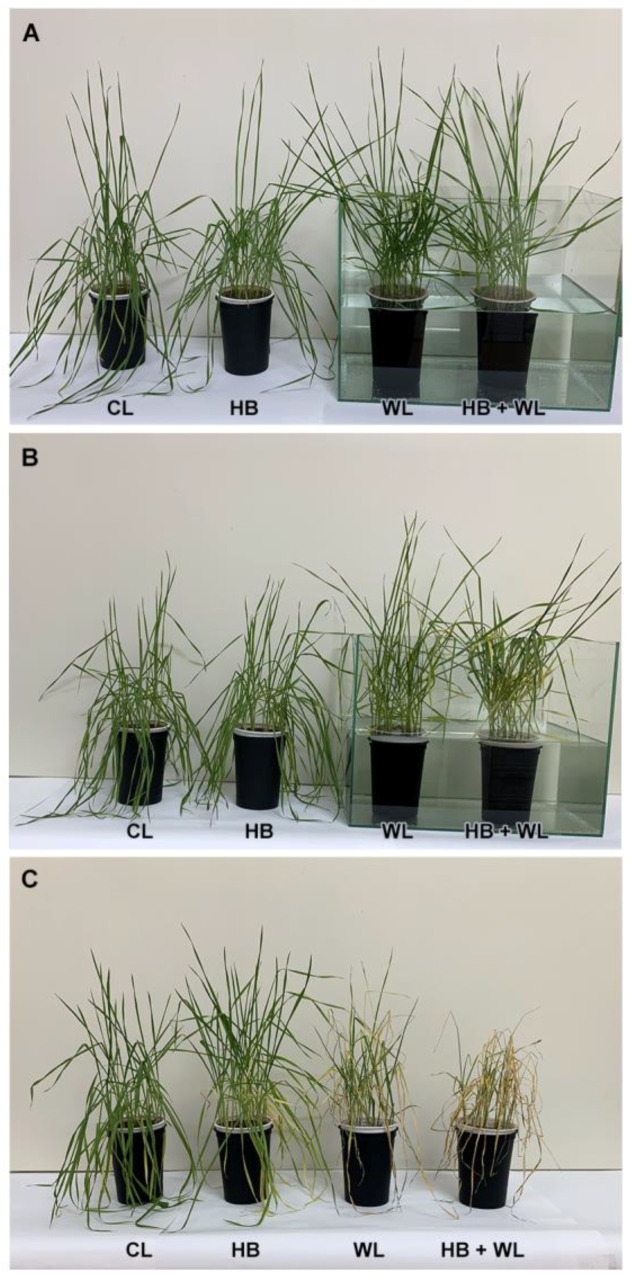
Phenotypic traits of wheat plants treated with herbicide and subjected to waterlogging: (**A**) 96 h of waterlogging, (**B**) 168 h of waterlogging, and (**C**) 96 h of recovery after restoring the normal irrigation regime. Treatments: CL—Control; HB—Herbicide; WL—Waterlogging; HB + WL—Herbicide + Waterlogging.

Phenotype alterations observed due to herbicide foliar application and consecutive waterlogging are presented in Figure 1. As expected, the herbicide did not alter wheat phenotype throughout the experiment. Initially (at 96 h of waterlogging), there were no obvious differences between treatments (Figure 1A). Obvious wilting and yellowing were observed in wheat plants subjected to waterlogging and herbicide + waterlogging after 168 h of stress (Figure 1B). These negative effects amplified after 96 h of recovery and appeared stronger for the plants treated with Serrate^®^ and subjected to subsequent waterlogging (Figure 1C).

### 2.2. Stress Markers Content

To assess the effects of herbicide and waterlogging stress, we measured the content of malondialdehyde, free proline, and hydrogen peroxide.

**Figure 2:**
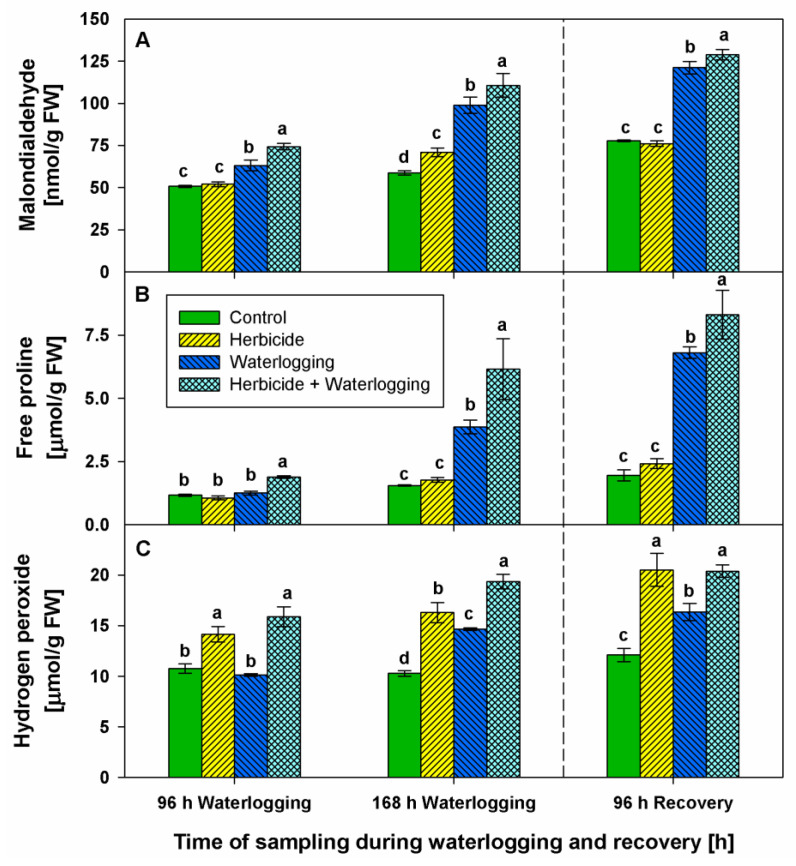
Content of malondialdehyde (**A**), free proline (**B**), and hydrogen peroxide (**C**) in the leaves of wheat treated with herbicide and exposed to waterlogging. Control values at 0 h: (**A**) 57.210 ± 4.104; (**B**) 0.837 ± 0.039; (**C**) 6.568 ± 0.696. Different letters within a group of columns indicate statistical significance between treatments at *p* < 0.05.

Malondialdehyde (MDA) content augmented temporarily by 20% (96 h of the stress program) in herbicide-treated plants (Figure 2A). Waterlogging progressively increased MDA levels over time (20%, 70%, and 80% above the control at 96 h, 168 h of stress, and after 96 h of recovery, respectively). When waterlogging was applied after herbicide treatment, MDA accumulated in higher degrees up to 50%, 90%, and 90% compared with controls at 96 h and 168 h of stress, and at 96 h of recovery, respectively. 

Serrate^®^ did not significantly alter proline concentration during the time of monitoring. In contrast, proline levels (Figure 2B) severely increased at 168 h of waterlogging (150%) and after 96 h of recovery (250%). The combined treatment further increased the stress marker at 96 h (60%) and 168 h (300%) of waterlogging, and after 96 h of recovery (330%).

Hydrogen peroxide concentrations (Figure 2C) increased gradually with time by 30% (at 96 h of the stress program), 60% (at 168 h of stress), and 70% (at 96 h of recovery) above the respective control after Serrate^®^ application. The content of H_2_O_2_ raised moderately (40%) at 168 h of waterlogging and after 96 h of recovery. The combined treatment increased H_2_O_2_ amounts by 50% and by 90% (at 96 and 168 h of waterlogging, respectively) and by 70% after 96 h of recovery.

### 2.3. Content of Non-Enzymatic Antioxidants 

The content of total phenolics and thiol-groups-containing compounds were measured as part of the non-enzymatic antioxidant defense system.

**Figure 3:**
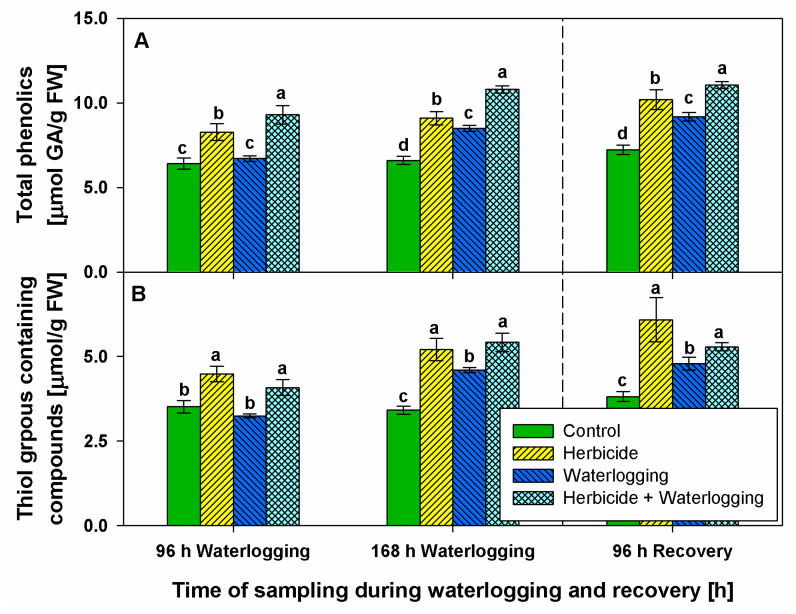
Content of total phenolics (**A**) and thiol-groups-containing compounds (**B**) in the leaves of wheat treated with herbicide and exposed to waterlogging. Control values at 0 h: (**A**) 4.557 ± 0.223; (**B**) 2.198 ± 0.115. GA—gallic acid. Different letters within a group of columns indicate statistical significance between treatments at *p* < 0.05.

Serrate^®^ permanently augmented phenolic levels (Figure 3A) by 30% (at 96 h of waterlogging) and by 40% (at 168 h of waterlogging and after 96 h of recovery). Waterlogging enhanced total phenolic levels up to 30% (at 168 h of stress and after 96 h of recovery). The combined treatment raised phenolic levels throughout the experiment by 50% (96 h of waterlogging), 60% (168 h of waterlogging) and 50% (96 h of recovery).

As expected, herbicide treatment permanently increased thiols up to 53% (at 168 h of waterlogging) and then to 60% (at 96 h of recovery) above the respective controls (Figure 3B). Thiols were significantly enhanced by waterlogging at 168 h of stress (35%) and at 96 h of recovery (26%). The combined treatment also resulted in the accumulation of thiol compounds at 96 h (16%) and 168 h (59%) of waterlogging, and after 96 h of recovery (40%), but the increase occurred to a smaller degree than after the herbicide treatment only.

### 2.4. Activity of Antioxidant Enzymes

The activities of key antioxidant enzymes such as catalase, guaiacol peroxidase, superoxide dismutase, and glutathione reductase were monitored to evaluate the plants’ responses to the treatments.

**Figure 4:**
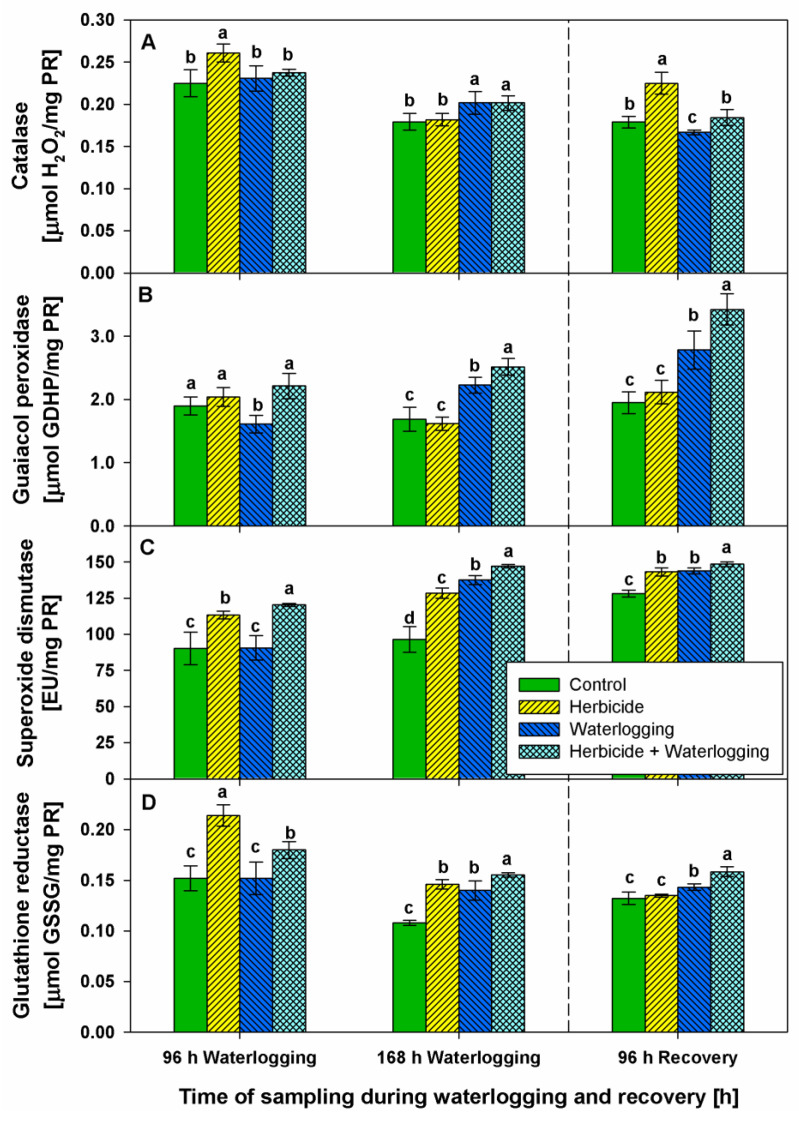
Activity of catalase (**A**), guaiacol peroxidase (**B**), superoxide dismutase (**C**), and glutathione reductase (**D**) in the leaves of wheat treated with herbicide and exposed to waterlogging. Control values at 0 h: (**A**) 0.211 ± 0.020; (**B**) 1.767 ± 0.192; (**C**) 60.280 ± 9.942; (**D**) 0.158 ± 0.012. GSSG—oxidized glutathione. Different letters within a group of columns indicate statistical significance between treatments at *p* < 0.05.

Herbicide provoked a slight rise in catalase activity (Figure 4A) at 96 h of waterlogging (20%) and after 96 h of recovery (30%). Waterlogging, as well as the combined treatment, did not significantly change catalase activity throughout the experimental period. 

Herbicide application did not significantly change guaiacol peroxidase (POX) activity throughout the experimental period (Figure 4B). Initially (at 96 h of stress), waterlogging slightly reduced POX activity (15%), but then gradually raised it up to 40% (at 96 h of recovery). The combined treatment progressively increased POX activity in a time-dependent manner by 20% (at 96 h of waterlogging), by 50% (at 168 h of waterlogging), and by 80% (at 96 h of recovery). Serrate^®^ increased superoxide dismutase (SOD) activity by 30% (at 96 and 168 h of stress), but after 96 h of recovery, reverted it to 10% above the respective control level (Figure 4C). Waterlogging increased SOD activity up to 40% (at 168 h of stress), but finally (at 96h of recovery) became 10% above the respective control. The combined treatment enhanced SOD activity in a time-dependent manner during stress up to 50% (at 168 h of waterlogging), but after 96 h of recovery, became 20% above the respective control.

As expected, the highest increase in glutathione reductase (GR) activity (40%) was found after Serrate^®^ treatment (96 h and 168 h after waterlogging), but at 96 h of recovery, it reached the respective control level (Figure 4D). Waterlogging enhanced GR activity by 30% (at 168 h of stress). The combined treatment raised GR activity slightly (20%) at the beginning and end of monitoring, and substantially (40%) at 168 h of stress. 

In general, waterlogging did not cause substantial alterations at the first measurement (96 h) on neither the phenotype nor the biochemical analyses, but the combined treatment did cause substantial alterations throughout the experiment.

## 3. Discussion

Usually, the instructions for the application of herbicides clearly state that herbicide use is not recommended on crops that are preliminarily subjected to different stressors, including waterlogging. To the best of our knowledge, there is limited information on how subsequent adverse environmental conditions affect the physiological traits of crop plants subjected to selective herbicide, which often occurs in the field. Recently, we documented the alterations in physiological responses of wheat seedlings to soil drought after the application of Serrate^®^ [20]. In order to enhance the knowledge of different types of water stress in the present study, we used a similar model system in which plants were treated with Serrate^®^ herbicide and, a few days later, were waterlogged. It was documented that the lack of sufficient O_2_ in waterlogged plants augments intracellular ROS (O_2_^•^¯, ^•^OH and H_2_O_2_), which provokes oxidative injury of cellular membranes (damaging the phospholipid layers) and macromolecules (DNA, proteins), and disrupts biomembranes, cell organelles, and tissues [6,18]. In addition to waterlogging, many herbicides applied alone may induce oxidative events or oxidative stress, which have been reviewed by [6,18,21]. Clodinafop-propagyl (herbicide TOPIK) was also reported to provoke oxidative stress when it was applied to leaf disks and intact 7-day-old wheat, rye, and maize plants [22]. The reaction was manifested by the induction of lipid peroxidation, superoxide anion generation, total antioxidant activity, and antioxidant enzymatic activity. The authors claimed that a protective antioxidant mechanism was provoked in rye and wheat due to the highest antioxidant enzymatic activation [22]. Our study expands upon the information about stress combinations applied in a consequent manner (waterlogging after foliar herbicide treatment).

The temporary MDA peak after herbicide application, together with the rise in H_2_O_2_, may indicate that Serrate^®^ led to transient oxidative events, but after recovery, lipid peroxidation decreased in wheat plants. In addition, wheat plants seemed to have a good phenotype status, verifying the wheat’s tolerance to foliar Serrate^®^ application. The MDA accumulation established in waterlogged plants increased with stress duration, which indicated impaired lipid peroxidation status of plants under waterlogging as documented earlier [15,16,23,24]. Hydrogen peroxide was also reported to amplify in response to waterlogging and corresponded well with the oxidative stress observed in different sensitive plants [23,25,26]. When waterlogging was applied sequentially to Serrate^®^, the amounts of lipid peroxidation product MDA were even higher. The other stress markers (H_2_O_2_ and proline) were altered in a similar manner, which indicates that wheat plants subjected to waterlogging, and especially to herbicide + waterlogging, cannot mitigate ROS-induced damages. The phenotype traits of wheat plants subjected to herbicide + waterlogging also indicated a worse physiological status then those observed after waterlogging. 

Proline is an important amino acid that has multiple roles. It participates in primary metabolism, and acts as an ROS scavenger, a compatible solute, an indicator of stress, etc. [27]. There is contradictory information on the fluctuation in proline concentration after flooding stress depending on plant species, and stress application and its duration. Accumulation [13,14,15,25,26,28] or reduction [17,23,26] in proline amounts were reported in different plant species. In the current study, the application of the selective herbicide did not alter proline concentration. Oppositely, simultaneous accumulation of both proline and MDA signifies that proline acts as a stress marker in wheat plants subjected to waterlogging, which was noticed more strongly after herbicide + waterlogging. The H_2_O_2_ increase might act as a signal molecule and induce plant defense mechanisms after xenobiotic application, which was reviewed by Hasanuzzaman et al. [18]. The observation of a good phenotype status in wheat plants after Serrate^®^ application corresponds well with the increased endogenous antioxidant defense (including enzymatic and non-enzymatic), which confirms wheat’s tolerance of this selective herbicide. Similar to our results, it was documented that clodinafop raised SOD activity but did not substantially change CAT activity [29]. Clodinafop-propargyl led to a dose-dependent increase in CAT activity [22] in wheat. In addition, waterlogging was reported to increase POX and CAT activities in different plant species: cherry rootstocks [25], spinach [26], barley [30,31], tomato [32], and peach [33].

The low-molecular-weight thiol compounds, known to mainly include glutathione [34,35] as well as GR, were substantially boosted after Serrate^®^ application. Apparently, glutathione may detoxify lipid peroxides, herbicides, and other molecules directly through conjugation or indirectly by redox signaling, glutathionylation (post-translational reversible protein modification), or changing DNA binding features [36,37,38]. Phenolic compounds are known to impede ROS overproduction due to their scavenger properties and are able to restrict lipid peroxidation [39]. Phenolics increased by herbicide application probably halt oxidative events and wheat plants are able to support good phenotype status. The induction of non-enzymatic antioxidant defense (especially glutathione) contributes to the beneficial response and survival under waterlogging [17,25]. On the contrary, we suggest that wheat plants subjected to waterlogging, and especially to herbicide + waterlogging, appear to lose the balance between ROS overproduction and defense systems, and are not able to alleviate the oxidative stress. It seems that the induction of enzymatic and non-enzymatic defense systems was insufficient for plant recovery. It is possible that the plants subjected to waterlogging and to herbicide + waterlogging became distressed because of the postponed or insufficient induction of thiol-containing compounds, phenolics, and activities of GPX, SOD, and GR in wheat. 

In general, the effect of the combined treatment was stronger than that of waterlogging even during recovery. It was reviewed earlier [40] that in the case of multiple stress agents, their cumulative action often far exceeds the effect of individual application (cross-synergism). Such cross-synergism was documented in herbicide-treated maize plants cultivated under low temperatures and high soil moisture [41]. The highest levels of stress markers (MDA, proline, and H_2_O_2_) observed after recovery in wheat subjected to herbicide + waterlogging suggest that Serrate^®^ acted synergistically with the subsequently applied waterlogging. It seems that the oxidative stress events continued even during the recovery witnessed by a gradual increase in stress markers and worsened phenotypic traits. 

## 4. Materials and Methods

### 4.1. Plant Material and Treatments

Seeds of wheat (*Triticum asetivum* L., cv. Sadovo-1) were purchased from the Institute of Plant Genetic Resources (Sadovo, Bulgaria). This variety is characterized by its high productivity, good tolerance to drought and low temperatures, and relatively resistance to lodging. Sadovo-1 is one of the most extensively grown wheat varieties in Bulgaria.

Soil grown wheat seedlings were sprayed with herbicide Serrate^®^ on the 17th day (2nd–3rd leaf phase) with 1 mg mL^−1^ of aqueous solution as suggested by the manufacturer’s provided information. Wheat seedlings were grown in a growth chamber (60% relative humidity, 22/19 °C and 16/8 h light during day/night). Waterlogging was performed 72 h after herbicide treatment and was implemented by transferring the pots into an external container filled with a water level 2 cm higher than the soil [31].

The stress program was ended seven days after the beginning and the stressed plants were transferred to normal irrigation conditions for recovery. The plant material was collected for analysis at 0, 96, and 168 h of waterlogging stress and after 96 h of recovery for the following variants:

1. Control plants, normally irrigated.
2. Plants treated with herbicide and normally irrigated.
3. Plants subjected to waterlogging for 7 days.
4. Plants treated with herbicide and subjected to subsequent waterlogging for 7 days.

### 4.2. Biochemical Analyses

The ground 4 mL of 0.1% cold trichloroacetic acid leaf material (approximately 250 mg) was centrifuged for 30 min at 15,000*× g* (4 °C). The supernatant was used to analyze the stress markers. The concentration of malondialdehyde as a marker of the levels of lipid peroxidation was measured according to the method of Kramer et al. [42]. The reaction mixture was incubated for 45 min at 100 °C and the absorbance was read at 532 nm and 600 nm. The content of MDA was calculated on the basis of the 155 mM·cm^−1^ extinction coefficient. The content of free proline in the samples was determined according to Bates et al. [43]. We incubated 0.5 mL of supernatant with ninhydrin reagent for 1 h at 100 °C. The absorbance was read at 520 nm and the concentration of proline was calculated by a standard curve prepared with known amounts of proline. The content of hydrogen peroxide was measured according to Alexieva et al. [44]. After a 1 h incubation of 75 mkl supernatant with equal amount of 1 M KI, the absorbance was read at 350 nm, and the concentration of H_2_O_2_ was calculated by a standard curve. The content of free-thiol-groups-containing compounds was determined by incubation of 40 mkl supernatant with 150 mkl Ellman’s reagent for 10 min at room temperature. The absorbance was read at 412 nm [45]. The total content of phenolic compounds was measured according to Swain and Goldstein [46]. The reaction mixture was incubated for 2 h at room temperature, then the absorbance was read at 725 nm and the content of total phenolics was calculated by a standard curve prepared with gallic acid. 

The activity of antioxidant enzymes was measured in supernatant obtained from approximately 200 mg of leaf material after grinding in 3 mL of cold 100 mM potassium phosphate buffer (pH 7.0) containing 1 mM EDTA and 1% PVP and centrifugation at 15,000*× g* (4 °C). The activity of guaiacol peroxidase (EC 1.11.1.7) was measured using 1% guaiacol as an electron donor and 15% H_2_O_2_ as a substrate. The change in the absorbance was monitored for 1 min at 470 nm [47]. Catalase activity (EC 1.11.1.6) was measured by following the decomposition of 6% H_2_O_2_ for 1 min at 412 nm [48]. Glutathione reductase activity was measured by monitoring the reduction of GSSG for 1 min at 412 nm [49]. Superoxide dismutase activity was determined by the rate of inhibition of the photochemical reduction of nitroblue tetrazolium. The reaction was monitored at 560 nm. One unit of SOD defined the amount of enzyme needed to cause 50% inhibition [50]. The content of soluble protein was determined with Bradford’s reagent [51]. 

The chemicals used in the analyses were purchased from local representative of Sigma-Aldrich, (Saint Louis, MO, USA) Serrate^®^ was purchased from a local representative of Syngenta (Basel, Switzerland). Spectrophotometric measurements were conducted using Multiskan Spectrum (Thermo Electron Corporation, Uusimaa, Finland) and Shimadzu UV-1601 (Shimadzu, Kyoto, Japan) spectrophotometers. Supernatants were centrifuged in a refrigerated Sigma 2-16K centrifuge (SciQuip, Newtown, UK).

### 4.3. Statistics

The experiments were conducted three times. Samples for analyses were collected in three replicates. The data are presented as mean values ± SE. Duncan’s multiple-range test was used to assess the significant differences between treatments at *p* < 0.05.

## 5. Conclusions

Serrate^®^ application increased antioxidant defense and did not worsen phenotype plant traits, suggesting wheat adaptation. Waterlogging gradually enhanced the stress markers’ content, had lag-time in antioxidant (enzymatic and non-enzymatic) induction, and wheat phenotype traits did not improve after the recovery period. Serrate^®^ application induced a synergistic response in wheat subjected to waterlogging by aggravating the phenotypic traits of plants and did not recover successfully after cessation of the stress program; therefore, a monitoring forecast for flooding is recommended before Serrate^®^ application to wheat as it may be unable to recover.

## Data Availability

Not applicable.
